# Navigating trial design in radiation dermatitis research: paths to improvement

**DOI:** 10.1007/s00520-024-08959-5

**Published:** 2024-10-29

**Authors:** Cas Stefaan Dejonckheere, Leonard Christopher Schmeel

**Affiliations:** Department of Radiation Oncology, University Hospital Bonn, Venusberg-Campus 1, 53127 Bonn, Germany

Acute radiation dermatitis (RD) is a common side effect of radiotherapy, with a high prevalence in patients with breast, and head and neck cancer [1]. Clinically, it is characterised by erythema, swelling, pruritus, pain, and dry or moist desquamation, and severe cases (with erosions and ulcerations) might require radiation treatment interruption, impairing tumour control outcomes [2]. This has become rare following recent advances in radiation treatment technique (e.g. intensity-modulated radiation therapy), fractionation regimens (e.g. hypofractionation), and patient positioning (e.g. prone irradiation in breast cancer patients with large breast size); however, even mild symptoms can majorly impact quality of life and self-image [2–4]. Despite ongoing research efforts, potent preventative and therapeutic options remain limited and treatment recommendations across different guidelines show considerable discordance, resulting in substantial variation in RD management amongst healthcare providers and institutions, relying on personal experience and opinions rather than evidence [5–7].

The recently published Multinational Association of Supportive Care in Cancer (MASCC) clinical practice guideline aimed to summarise the evidence regarding prevention and management of acute RD [8]. Following a four-round Delphi consensus process amongst 42 international experts, based on a comprehensive and systematic review of all evidence in the existing medical literature until September 2020 (235 original publications, of which 149 randomised trials), a recommendation (defined as at least 75% consensus) could only be reached for seven interventions: photobiomodulation therapy, Mepitel film, Hydrofilm, mometasone, betamethasone, olive oil, and Mepilex Lite (management only).

Despite the abundance of trials showing promising initial results, only few RD interventions prove useful in adequately powered and well-designed trials [9]. For the majority of tested interventions, evidence is either lacking, insufficient, or even conflicting, leading to a very limited number reaching a high level of evidence. Reasons for this discrepancy are manifold, but mostly related to methodological limitations of the individual trials. Some ways to improve clinical trial design in radiation dermatitis research have been identified and are discussed below:

- Aim for a homogeneous patient collective in terms of patient (treatment site, type of surgery) and treatment (prescribed dose and fractionation, concomitant therapies) characteristics and report these data appropriately;
- Consider established risk factors for RD development and stratify patients accordingly: skin type (e.g. Fitzpatrick scale), breast volume in breast cancer (either cup size or planning target volume), radiation treatment characteristics (technique, dose and fractionation regimen, boost, bolus) [10, 11];
- Randomise patients between the intervention that is being investigated and an adequately defined standard of care arm, ideally receiving an inactive placebo control or identical vehicle. Consider intrapatient randomisation (e.g. between breast halves or neck sides), as it omits the need for the above-mentioned stratification and thus limits confounding in outcome assessments. This has been successfully used in previous trial on this topic [12, 13];
- Regularly check for compliance and appropriate use of the intervention and encourage patients not to use any other complementary topical products that might influence outcome;
- Define clear endpoints and (in trials investigating prophylactic interventions) discuss the actions taken to manage these accordingly (especially the use of other topical antibiotics or corticosteroids);
- Limit bias by integrating single or double blinding in outcome assessment, especially if subjective clinician-reported outcomes (CROs) are reported;
- Include the patient’s perspective by making use of radiation-related patient-reported outcomes (PROs), as significant discrepancies with CROs exist in the context of RD, with the latter failing to capture the impact on quality of life [14, 15]. Validated instruments in the context of RD are available, e.g. the Radiation-Induced Skin Reaction Assessment Scale or Skindex-16 [16, 17];
- Consider the use of biophysical parameters to objectively assess outcome and improve the robustness of the data, as these are not subject to inter- and intra-rater variability. Validated non-invasive instruments in the context of RD include reflectance spectrophotometry, transepidermal water loss, or cutaneous blood flow measurements [10, 18–20].

The above-mentioned suggestions are derived from previously conducted trials on RD management that yielded overall high levels of consensus. These steps might guide future research and serve as a starting point for study conception and design. Uniform trials facilitate a direct comparison between interventions, expedite recommendations, and might turn the exponential increase of publications into an increase of preventative and therapeutic options for RD, ultimately improving patient care.
